# Access to rehabilitation: patient perceptions of inequalities in access to specialty pain rehabilitation from a gender and intersectional perspective

**DOI:** 10.3402/gha.v9.31542

**Published:** 2016-08-26

**Authors:** Maria Wiklund, Anncristine Fjellman-Wiklund, Britt-Marie Stålnacke, Anne Hammarström, Arja Lehti

**Affiliations:** 1 Department of Community Medicine and Rehabilitation, Physiotherapy, Umeå University, Umeå, Sweden; 2 Umeå Centre for Gender Studies in Medicine, Umeå University, Umeå, Sweden; 3 Department of Public Health and Clinical Medicine, Social Medicine, Umeå University, Umeå, Sweden; 4 Department of Community Medicine and Rehabilitation, Rehabilitation Medicine, Umeå University, Umeå, Sweden; 5 Department of Clinical Sciences, Professional Development, Umeå University, Umeå, Sweden

**Keywords:** chronic pain, treatment of pain, multimodal rehabilitation, gender bias, equity in health, intersectionality, qualitative interviews, Sweden

## Abstract

**Background:**

Long-term musculoskeletal pain is common, particularly among women. Pain conditions are a concern in primary health care, and people with severe and complex pain are referred to specialty health care. There is gender bias in access, counselling, assessment, and treatment of long-term pain.

**Objective:**

This study explores patient accounts and perceptions about important (social) factors for accessing specialised pain rehabilitation from gender and intersectional equality perspectives. We aimed to identify potential biases and inequalities in accessing rehabilitation resources at a specialised rehabilitation clinic.

**Design:**

Individual semi-structured interviews were conducted with 10 adults after an assessment or completion of a specialised rehabilitation programme in northern Sweden. Qualitative content analysis was used to explore patients’ perceptions of important factors for accessing rehabilitation.

**Results:**

One main theme was formulated as *Access to rehab – not a given*. Three categories of perceived inequality were demonstrated: power of gender, power of social status, and power of diagnosis. Participants perceived rehabilitation as a resource that is not equally available, but dependent on factors such as gender, socio-economic status, ability to work, ethnicity, or age, and more subtle aspects of social status and habitus (e.g. appearance, fitness, and weight). The character of diagnosis received (medical versus psychiatric or social) was also noted.

**Conclusions:**

It is crucial that professionals are aware of how potential inequalities related to gender, social status, and diagnosis, and their intersections, can be created, perceived, and have influence on the processes of assessment and treatment. Reduction of social determinants of health and biases remain important within global, national, and local contexts.

## Introduction

Long-term musculoskeletal pain is common worldwide, occurring in approximately 19% of the adult European population. Women seem to be affected more frequently (1). Long-lasting, benign pain is influenced by, and interacts with, physical, emotional, psychological, and social factors. Therefore, a biopsychosocial perspective is required in order to understand and manage long-term pain (2, 3). Patients describe a range of negative consequences of pain, including significant pain intensity, psychological distress, anxiety and depression, insomnia, reduced ability to work (sick leave), ill health, and low quality of life (4–6). Individuals suffering from long-term pain may experience mental distress and may encounter social stigma, shame, or discrimination because of their condition (7–12). In addition, such pain is occasionally referred to as a ‘medically unexplained’ condition. This may be problematic for those suffering from pain (7, 13). Having a medically unexplained illness may be particularly troublesome in a health insurance system where having a specific medical diagnosis is an advantage in qualifying for sickness benefits. Public health insurance was a former cornerstone of the Swedish welfare state. In recent years, the phenomenon of individuals being excluded from sickness benefit has been the subject of heated debate among both lay people and politicians.

In welfare states, such as in Sweden, the primary healthcare system is often primarily responsible for treatment and decisions about eventual sick leave (14). Pain conditions are a primary healthcare concern in Sweden, because 20–40% of people seeking primary health care suffer from pain conditions (15). Patients with recurrent, widespread, or severe pain can be referred to multidisciplinary assessment and multimodal rehabilitation in specialty health care. In terms of treatment models, multimodal pain rehabilitation has proven successful for patients with complex long-term pain (12, 14, 16). However, not all individuals who undergo assessment by a specialty team are selected for the multimodal rehabilitation programme, because they may need further investigation or benefit from unimodal treatment. Those who are not selected may find this hard to understand and accept.

Medical researchers point to gender bias in access, counselling, assessment, and treatment of long-term pain in primary and specialty care (17–20). For instance, women with lower levels of education are less often selected for rehabilitation programmes (19, 20). Bias in health care is defined as unintended or systematic neglect of certain groups. This could be because of socio-economic position, educational level, sex/gender, or age (17, 21–23). Intersectional perspectives are therefore crucial to a better understanding of people with conditions such as musculoskeletal pain (8, 24, 25). Feminist perspectives point out the impact of social and cultural variables on mental health and emphasise gender inequalities and intersections of power differentials as key factors in women's emotional distress (26) and pain (19).

Our theoretical frame is informed by the view of gender as a central social category and a social construction that is present in all social relationships, social structures, and power hierarchies, as well as in various health and illness processes (27–29). Gender is defined as a social determinant of health (30), which intersects with other aspects of oppression and social stratification (8, 31). Using Bourdieu's conceptualisation (32, 33), health care and rehabilitation are viewed as ‘fields’ where gendered and intersectional processes come into play, and where social status is continuously created by professionals and patients, depending on their social and symbolic ‘capital’ and ‘habitus’ (34). From the perspective of Connell (28), health care as societal institution may reflect gender orders and gender relations in the overall society. We may also find certain ‘gender regimens’ within this specific sector, for example, connected to rehabilitation practices (34).

Little is currently known about the selection processes for specialised pain rehabilitation from an intersectional equality and gender perspective. To our knowledge, few studies have explored inequalities in assessment or pain rehabilitation from the patient's perspective. In a research project about equal care in pain rehabilitation, we investigated various aspects of gendered and social processes (19, 20, 35–37). Further identification of potential biases and inequalities in accessing rehabilitation resources, as perceived by individuals seeking help for long-term pain at a specialised rehabilitation clinic, is crucial. In this interview study, we aim to explore patients’ accounts and perceptions about important (social) factors in accessing specialty pain rehabilitation from a gender and intersectional equality perspective.

## Methods

### Setting and design

This study was part of an overarching research project in northern Sweden named Equal care in rehabilitation, which combines quantitative (19, 20, 36) and qualitative methods (35, 37). In this substudy, we used a qualitative approach (38, 39) to focus on and deepen our understanding of patients’ perceptions and experiences. This specific focus distinguishes the present analysis from a previous one in which patients’ voices were merged with the views of professionals to illustrate the path of suffering (Via Dolorosa) along the normative chain of rehabilitation from primary care to the specialty pain clinic (37). Individual interviews were analysed by qualitative content analysis as applied in health sciences research (40). Qualitative content analysis is a suitable method for analysing human communication and individuals’ experiences in a systematic way (41) and has been used to explore the meanings of gender and ethnicity (42–44).

The interview study was conducted in a clinical setting. Patients were referred by general practitioners for assessment (and eventual participation in a multimodal rehabilitation programme) at a specialty pain rehabilitation clinic in northern Sweden. Inclusion criteria for the multimodal programme were: the presence of disabling, non-malignant, long-lasting, and complex musculoskeletal pain (causing absenteeism due to sickness or major interference in daily life); aged 18–65 years; and not in need of further medical investigation. Thus, not every patient was selected for the programme. Those who were not selected were referred back to primary care with a rehabilitation plan.

### Procedures and participants

Participants were purposively sampled from among individuals assessed recently at the pain rehabilitation clinic. They could have been selected for the multimodal programme or referred back to primary care. The sampling strategy aimed for variation in perceptions and experiences, and therefore, patients of different ages, sexes/genders, socio-economic, and ethnic backgrounds were invited to take part. The inclusion criterion for this study was suffering from severe and complex benign, long-term musculoskeletal pain. Fluency in Swedish was an inclusion criterion for attending the multimodal rehabilitation programme (partly based on group sessions), and therefore also for being invited to this study. Exclusion criteria were diagnosis of other pain than benign musculoskeletal pain. One of the authors (B-MS, the responsible physician) made the selections to attain diversity of ages, gender, and country of birth. The participants were first contacted with a letter signed by the responsible physician, and then a research assistant contacted them by telephone.

Between December 2010 and February 2011, 10 patients (5 women and 5 men) aged 35–65 were eligible and interviewed. Of these, four were born in Sweden, three in Finland, and three outside Europe (Middle East or Latin America). Half of the participants had a mother tongue other than Swedish, but all spoke Swedish fluently. Five participants had higher education, and five did not have education beyond compulsory school. Four were working full-time and six were on sick leave or unemployed. Eight participants had undergone assessment at the rehabilitation clinic but not been selected to participate in the multimodal programme. Two had undergone assessment and had been selected to participate in the rehabilitation programme, which they had done.

### Data collection

Individual, semi-structured, qualitative interviews were conducted (38, 39). An interview guide was used to support the interviews. The guide was based on open-ended questions about patient perceptions and experiences of assessment at the rehabilitation clinic, and participation in the rehabilitation programme. Aspects of gender, ethnicity, and class were of particular interest, but other aspects of potential perceived inequality were also explored with probing and follow-up questions. Examples of questions in the interview guide are: ‘How was your opportunity to make your voice heard in the meeting with the professionals at the specialty rehab?’ ‘What are your associations when you hear the expression “care on equal terms” [as it relates to your personal experience]?’, ‘Did you perceive that the assessment [at the specialty clinic] was influenced by your sex/gender, education, country of birth, or other characteristics?,’ and ‘What happened after the assessment?’ All interviews were digitally recorded and transcribed verbatim.

### Data analyses

Data analyses were conducted using qualitative content analysis according to Graneheim and Lundman (40). Our interpretative frame was informed by our specific focus on aspects of gender and equality. The initial inductive phase kept relatively close to patients’ own accounts and the manifest content (45). The later steps of abstraction were guided by our clinical and theoretical awareness of potential inequalities and biases linked to gender, class, age, or ethnicity. First, the interviews were read by each author individually (MW, AF-W, AL) with respect to our naïve understanding and a sense for the whole. Preliminary aspects that met the research aim were thoroughly explored and discussed. Initially, the whole material was divided into two content areas: experiences from primary care versus experiences from specialty rehab. The present analysis is limited to experiences from the specialty rehab. Experiences from primary care have been published elsewhere (37). The analysis was followed by detailed reading and coding on a slightly higher level of abstraction; this stage of the coding was done using qualitative software. Codes similar in content were then grouped into categories that corresponded to the research aim and our overall interpretation of the interviews. Finally, one main theme was formulated that captured the latent meaning, running as a red thread, throughout the codes and categories.

During the final steps of the analytical procedure, triangulation between researchers (all five authors) with different clinical or theoretical perspectives was used as a method to increase trustworthiness (38, 46). The iteration between codes and interviews was another way to ensure trustworthiness.

## Results

The results are presented as one theme: Access to rehab – not a given, which runs as a thread through the three categories: the power of gender, the power of social status, and the power of diagnosis (Table 1). Each category, with its interrelated subcategories, represents a factor of importance for accessing rehabilitation resources, as perceived by patients.

**Table 1:** Patient perceptions of access to rehabilitation presented as subcategories, categories and the main theme

Subcategories	Categories	Theme
Women and men are (not) different	The power of gender	
Men's voices are heard and get through		
Women with pain are drowned out in the crowd		
Patients are treated differently	The power of social status	Access to rehab – not a given
Suit and tie helps		
Money talks		
Diagnosis with (non)status	The power of diagnosis	
Diagnosis and ability to work as ‘entrance ticket’		
Unfair assignment of diagnoses		

### Access to rehab – not a given

The theme, Access to rehab – not a given, captures the view of rehabilitation as a resource that is not equally available to all who need it. The participants were aware that they would not automatically qualify for rehabilitation and support for coping with pain. They articulated their own or others’ experiences of inequality, either in connection to visiting the pain clinic or in a more general sense. The type of problem, diagnosis, ability to work, gender, ethnicity, and social status were factors perceived as either facilitating or hindering access to rehabilitation resources and professional support. Notably, overlapping category content points to intersections between social aspects.

#### The power of gender

The category ‘the power of gender’ consists of the subcategories: women and men are (not) different, men's voices are heard and get through, and women with pain are drowned out in the crowd. The category demonstrates how gender perceptions permeated participant beliefs about treatment and access to rehabilitation, although not every participant had previously reflected upon these issues.

##### Women and men are (not) different

Some participants stated that women and men are different in terms of genetics, life-situations, and symptoms. Consequently, they thought these factors should be taken into account during assessment and treatment, as well as during decisions about rehabilitation programme admission. One woman stated that men's and women's situations are different because a woman is expected to be an ‘octopus’ with four pairs of hands. She was disappointed and thought she had been treated unfairly because she presented herself as ‘capable and quick’ during the assessment. Despite many years of suffering with pain, psychological trauma, and social pressures, this was how she behaved. And in her view, the facade of ‘capability’ was both part of her coping strategy and at the heart of her problem. She thought this (presenting appearance) should have been a strong reason for inclusion in the programme; whereas she thought the professionals saw it as a reason for exclusion.

Others did not relate their experiences of being poorly treated at the rehabilitation clinic as an issue of gender or gender inequality; rather, they framed the issue as a question of being able to demonstrate strength and to provide answers:

Researcher: You said [earlier] *that you are very* ‘*thick-skinned’ and would speak up if you felt badly treated*. *Do you think that this is related to your sex, being a man*?

Participant: Hmm … I don't know. There are many men with low self-esteem, so … perhaps there are a greater percentage of men with thick skin, but there are also many tough girls who dare to speak their minds, so I don't know ….

##### Men's voices are heard and get through

Being seen, heard, and respected was an important issue to participants. Some expressed the view that it was easier for men to make their voices heard and have smoother passage through the healthcare system because they were regarded as more assertive. Women were regarded as being more in need of the support and advocacy of someone who ‘brought action’, and this was preferably a man.

Researcher: Do you think that people receive different care?

Participant: Hmmm, depending on the age, and sex, and what sort of person one is.…

Researcher: Did you feel that the response you got was affected by you being a woman?

Participant: Maybe not just because I'm a woman … but maybe if you aren't the type of person who just walks in there and claims that ‘I need this’, but you are probably the type of person that says ‘no, but this will pass’ … It's almost as if you need to bring someone else (with you).

Researcher: In order to be taken seriously?

Participant: Yes, and then most preferably [that person will be] a man, or someone who can speak well.

On the question of whether she thought she would have got different treatment if she had been a man, the participant emphasised that women are expected to take more responsibility, and to manage on their own:

Participant: I actually think that if it had been a man who had pain, workwise, he probably would not have been expected to arrange everything himself. I arranged everything myself.

The perceptions that men have easier access to rehabilitation resources, receive more support, or have less responsibility may reflect dimensions of general bias and inequity related to gender. Other aspects of ‘having a voice’ or ‘lacking a voice’ such as not being a native Swedish speaker were also raised.

##### Women with pain are drowned out in the crowd

This subcategory of women with pain are drowned out in the crowd reflects the women's sense of invisibility and having less value or recognition because they were women. The subcategory also refers to intersects between the power of (male) sex/gender and power of certain (medical and specific) diagnoses. Other intersects were apparent, for instance, intersections of ethnicity (Swedish), sex/gender (woman), and diagnosis (fibromyalgia). The experience of marginalisation and low status throughout the rehabilitation process was expressed as ‘Swedish women with pain are treated like they are on an assembly line’.

In contrast, another woman, whose pain started in connection to an accident, had a more positive experience during her rehabilitation programme treatment. She felt her voice was heard, and that the rehabilitation personnel were on ‘her side’. Generally, participants regarded women patients as less valued and holding a lower status than men within the healthcare system.

#### The power of social status

‘The power of social status’ category is based upon the subcategories: patients are treated differently, suit and tie helps, and money talks. The category reflects perceptions of inequities related to socio-economic status, class, gender, and ethnicity. To some extent, this category represents general perceptions and is not related to the specific clinic, assessment, or programme where the participants received care.

##### Patients are treated differently

A common opinion was that patients are treated differently by health professionals, based on factors such as economic status, profession, age, nationality, and sex/gender. Aspects of appearance, weight, and fitness were mentioned by some participants. Perceived unfairness was commonly related as others’ experiences. For example, one woman said that age and ethnicity matters with reference to her parents-in-law, who were born in Finland: ‘They do not ask questions of those with higher education because the language makes it difficult to express themselves’. The same woman stated that, ‘Everything costs money in health care, even referral to radiology’. She thought this was an explanation for why treatments were not made available to everyone (i.e. the cost made some people less worthy of treatment). She was not accepted in the rehabilitation programme and tried to cope with her pain on her own (using walks, warm pillows, and an acupressure mat).

Another participant, who had a low-status job in personal care, thought that people were treated differently because of their age, sex, and societal status. This was exemplified by her belief that her friend (a top administrator in the healthcare sector) received better treatment than she had. She also thought that her husband received a better response and more respect from the healthcare personnel than she did. Aspects connected to ethnicity, the advantage or disadvantage of being born in or outside Sweden were also expressed:

Participant: I can imagine that there are some cultures where the difference is very large [comparing Swedes and other ethnicities in terms of behaviour and attitudes], and there is a lot of ignorance among Swedes that affects healthcare encounters. To me, they [healthcare professionals] start to speak English at once, even though I've lived here since I was 17, and I'm 48 years old tomorrow.

##### Suit and tie helps (to access rehabilitation)

Participants remarked that men with higher social status (white-collar workers) had advantages in accessing assessment and rehabilitation, and were given more respect. Social constructions of gender, for example, being a man or a woman, also were important for patients. As one participant expressed it, ‘a suit and tie helps’ in contacts with the rehabilitation team.

Participant: One may be treated differently if you come in a suit and tie, so it matters if I arrive scruffy, or if I dress in a tie.

Women who dressed in ‘make-up and high heels’ were seen to have an advantage. However, this dress code was not always seen as a guarantee for being respected or having one's pain taken seriously:

Participant: Sometimes you go to a doctor when you have put on a little extra makeup and done your hair nicely. Then they [doctors] think you look great, and think ‘How can you have so much pain?’ – it isn't visible.

This subcategory points to participants’ awareness of the importance of being an ‘ideal’ and ‘respectable’ patient with some social power and status (habitus), expressed through status markers and attributes such as appearance, proper clothing, and moderate weight. This is the power of a fit and young body.

##### Money talks

The above metaphor of suit and tie can also represent differences in socio-economic status. Certain, often expensive, treatments were talked about as not always being available to those with less money, or as available only when working for a large company with generous occupational health insurance:

Participant: It is clear that those who have good jobs and earn good money … receive better care.

Participant: There are some physiotherapists who are good at treating fibromyalgia, but it costs so damn much, you cannot afford it.

*The power of social status*, including the metaphors of ‘suit and tie’ and ‘make-up and high heels’ that work as ‘entrance tickets’ to rehabilitation points to an intersection of gender, socio-economic, and educational status, and their impact on perceived inequalities in access to health care. The power of social status also points to subtle biases that are tied to appearance, including the power of a fit and young body.

#### The power of diagnosis

‘The power of diagnosis’ category consists of the subcategories: diagnoses with (non)status, diagnosis and ability to work as ‘entrance ticket’, and unfair assignment of diagnoses. Participants recognised certain diagnoses with interrelated aetiologies, preferably specific medical diagnoses, as crucial ‘entrance tickets’ to care and rehabilitation. They also viewed ability to work, that is, a good prognosis for rehabilitation back into working life, as facilitating access to support and care. Perceived inequalities in assessment and treatment, or unclear and unfair distribution of diagnoses, generated feelings of powerlessness and frustration.

##### Diagnosis with (non)status

The subcategory diagnosis with (non)status points to the participants’ awareness of differences in the status of certain diagnoses, and how this status moderated access to rehabilitation. Musculoskeletal diagnoses were generally preferred. Diagnoses connected to the ‘psyche’ or ‘social’, along with non-specific, diffuse symptoms or long-term pain, were thought to have a lower status. A treatment recommendation of ‘psych drugs’ was seen as demeaning. The experience of being demeaned and ignored because of non-specific or non-measurable pain problems was expressed as: ‘Who cares about the ones with pain?’ or ‘pain cannot be measured, and pain problems are disregarded’.

Biomedical health problems and diseases, which are possible to measure and treat, were felt to have a higher status. Fibromyalgia was one example of a ‘psychosomatic disease’ that was viewed as getting less attention and having a low reputation in health care. This view was put forward by a woman born in Latin America. The psychosocial-oriented approach used by the rehabilitation team was questioned. A man who suffered from pain and had a very high intake of pain medications expressed his disappointment and powerlessness:

Participant: The social parts [of assessment in rehabiliation] were alright – it was not that. I should not complain, but I did not come here to get the ‘socialization’ [Swedish: social träning] or something … I had hoped when I was sent here that they would do something instead of just talking … I am still on so much medication.

As seen above, those who were frustrated had expected more help than ‘just talk’. Consequently, some had difficulty accepting the assessment outcome, particularly if they were not offered participation in the rehabilitation programme.

Feelings of not being heard, believed, or minimised when meeting the interdisciplinary team were expressed by the men who were not selected for the programme, and who therefore had to continue rehabilitation training on their own:

Participant: The individual meetings with the physiotherapist, doctor, and social worker were positive, but when all four of us sat together I was disappointed. You get the feeling that they sit there and think that you are making things up and lying. I left not much the wiser. They could not really say what it was. I felt that I was the one who was leading the whole discussion, and it should not be that way … They said: ‘Keep working out, but remember to cut down a bit, perhaps’. They shouldn't just send me home to ‘try and rehab yourself’. Instead, they should either send me to a real specialist or they [the rehab clinic] should continue (working with me).

Participant: I felt that one of the team tried to minimize my problems. I thought that was really bad. His assessment was a bit different [compared to the others in the team]. But it has not had any [negative] consequences for me; it's Neurorehabilitation's assessment that is decisive anyway. We were not on the same wavelength; I didn't really understand. He wrote out the medication prescription and told me to rest while I exercise. You have to experiment, yourself, with your medicine to find some kind of acceptable level.

Others expressed how pleased they were with their treatment and emphasised that the team was ‘very professional’. One of the men from the Middle East, who participated in the programme, was very pleased because ‘… they made me relax. Even the psychologist and social worker were professional. They wanted to look at it [the problem] from all sides and angles, investigate my real concerns. It was really good’.

##### Diagnosis and ability to work as ‘entrance ticket’

Besides the importance of receiving a relevant explanation of symptoms and problems, receiving a diagnosis was seen as facilitating further access to specific examinations, radiology, or surgery, as well as to wider societal welfare resources such as sick leave and sickness allowance. Ability to work was seen as a prerequisite for rehabilitation and support. In those cases, the visit to the rehabilitation clinic did not lead to the expected diagnosis, dashed hopes, disappointment, and harsh consequences were described:

Researcher: … So you don't believe that the visit to pain-rehab helped, or was consistent with your expectations?

Participant: No, it is the opposite … in all the other places I have been, I have had hopes of getting help to find a way to feel better – and that possibility was shut down at pain-rehab because their focus had changed and become something entirely different. So it felt bad. You felt terribly alone in the situation, which is similar as it is in society now – everywhere it's just up to you to try and feel better. There is no one that can help me, so it feels lonely.

As seen in the quote above, the participant compared her experiences to her perception of managing alone in the wider society. On the question of whether there was something with which she was particularly dissatisfied, she answered:

Yes, well, with the decisions they made, after this visit I am now shut out from the health insurance system. Thus, the decision that they made, or the assessment they made, has not actually given me something, but it has instead made my situation worse. Economically … so that's … what I think is negative. In other words, … I actually went there in the hope of getting better and in the hope there was a program that would help. And instead they said ‘but we have no role here other than focusing on the labour market’. Oh, and it was as if to say ‘I'm not ready for that’ … and then end up in an investigation at the primary care health centre, and then one is excluded from sickness benefit. So their judgement/assessment of me has clearly influenced me negatively.

The disappointed participant understands the decision as related to recent changes in the ‘political system’ (the Swedish social security net), where the ability and prognosis of being able to go back into work is a stimulus for access to social support; or, as she puts it, ‘there is nowhere I fit in without the ability to work’. Another woman with a high educational level, who was born in Finland, was also disappointed and dissatisfied because she did not receive a diagnosis. Because of this, she was excluded from sickness benefit by the health insurance office.

##### Unfair assignment of diagnoses

Thus, the actual assignment of diagnoses and treatment was understood by the participants to sometimes be unfair and to result in inequity. For instance, one man from the Middle East felt powerless about his situation and questioned why he was not offered surgery for his intervertebral disk displacement. He felt it was strange and unfair when he compared his situation and symptoms to those of a female Swedish colleague who got surgery and pain relief:

Researcher: What could the best medical care do for you?

Participant: A work colleague, she got surgery, and she had exactly the same problem. Why could they not give me surgery? I never really got any answer.

Researcher: Was it something you were talking about?

Participant: Yes, I said I wanted surgery. They said ‘Yes, but we do not think surgery is needed’. I think they mentioned something about it being hereditary or that my back is worn out … but I do not believe in heredity.

Researcher: Is there anything that could help to get rid of or reduce the pain?

Participant: I hope that there is a way, whether it is surgery or blocking nerves or something that can be done to at least reduce the pain. So that I can use less analgesics, because I am increasing the dose all the time. Where will it end otherwise?

The above quote shows that the man had not understood or accepted the explanations about the nature of his pain that were given to him by the rehabilitation clinic team, and that he felt he was left alone with his questions, overwhelming pain, and need of medication. From his viewpoint, it was problematic and frustrating not to be selected for surgery or guided in pain management and medication adjustment.

As participants viewed it, receiving a specific diagnosis represented an ‘entrance ticket’ to care, rehabilitation, and welfare, as well as to social acceptance, confirmation, and social legitimacy. The diagnosis and rehabilitation procedure can be understood as both gendered and classed, and as such intersects with social aspects of gender, class, age, and ability to work.

Participants’ narratives point to the importance of getting a relevant and creditable diagnosis as a way to understand the pain and its causes, get relief from pain and suffering, be socially accepted and confirmed, access other examinations or treatments, and as a means to access support from the social health insurance system.

## Discussion

According to our participants, access to rehabilitation depends on the power of social factors such as gender, ethnicity, social status, and the variable status of certain diagnoses. The ability to work was seen as a prerequisite for rehabilitation. Subtler aspects were also revealed. Examples of these are appearance, fitness, and weight. The theme ‘Access to rehab – not a given’ points to participants’ perceptions of rehabilitation as a resource that is not equally available to everyone. This result is congruent with our earlier findings from a register study in the same setting (19, 20, 36).

Participants generally regarded women patients as being less valued and holding a lower status in the healthcare system than men. Women were regarded as being ‘in need of someone [preferably a man] who brought their case’, whereas men were regarded as having a stronger voice and the power to access more resources and support. Women were also viewed as having to take on greater responsibility, receiving less support, and being somewhat invisible in the system. This was formulated as women with pain are drowned out in the crowd. A number of studies suggest that physicians’ gendered expectations create gender differences in medicine. An example of this is found in neck pain rehabilitation (17, 47). Another example is found in a study of gender bias in general physicians’ initial examination of chest pain in men and women. The study found an overuse of services such as exercise testing and hospital care by men compared to women (23). In a recent study, Hammarström et al. (19) found that interdisciplinary teams in specialty health care may discriminate against poorly educated women with long-lasting disabling pain, but further research is still needed. Congruent with our view, Ahlsen et al. (48) argue that professionals in pain rehabilitation need to understand how constructs of gender interact with the pain story. With a better understanding of gender, a professional may help patients improve their health.

Receiving a diagnosis or explanation was important to participants in making sense of their pain. This finding is supported by other studies (49, 50). Not receiving a diagnosis or an understandable explanation (particularly if not selected for the rehabilitation programme) was tied to perceptions of inequality or unfair treatment. Receiving a diagnosis can enhance a patient's sense of legitimacy when suffering from an illness that can otherwise be viewed as non-medical, psychiatric, or social and thus stigmatised. Within a strict biomedical discourse, the ‘legitimate user’ of health care is defined as one who has a ‘genuine condition’ (21). In such a context, it is understandable that a ‘proper’ diagnosis (i.e. medical and specific) that proves legitimacy is crucial to patients (7, 9, 10, 49). Participants felt that receiving a diagnosis facilitated access to rehabilitation resources, as well as to wider societal resources such as sickness benefit. This supports the idea of diagnoses as both categories and processes, and points to the multilayered complexities of diagnosis (51).

The social construction of a ‘legitimate’ healthcare user, in this case of rehabilitation, can be defined as gendered, with the ideal masculine subject position constructed as an ‘infrequent-user’ and the feminine subject position as a ‘regular-user’ (21). By framing their condition within a biomedical discourse as opposed to ‘psychological’ or ‘social’, men (and perhaps women) can maintain a socially accepted identity (21). These gendered subject positions would be of great interest for further investigation in relation to rehabilitation and help-seeking. Such investigation should include patient experiences of legitimacy and respect because suffering from long-term pain is tied to complex gendered positions and expectations (7, 52).

From a theoretical standpoint, our results show perceived inequalities and a biased social and gendered selection process that can be understood with the help of Bourdieu's concepts of social and cultural capital that are reflected in individuals’ (gendered) habitus and expressed through appearance, habits, and behaviours (32, 33). The category the power of social status captures the intersection between gender, socio-economic position, body shape (weight and fitness), appearance, and in some respects age and ethnicity. The category points to how participants wish to be seen as credible. When identifying inequities in health care and rehabilitation, it is thus crucial to be aware of the intersection of social categories and constructions. Using a tool that addresses gender inequality, and intersecting social aspects, in daily clinical work may be one way of raising awareness (36). The tool can be included in clinical assessment of patients and as an instrument for critical reflection on gender bias during health professional team education.

Our intersectional study approach makes it clear that interplays between various social factors were created in a specific context and situation (53), and possibly a specific gender regimen (28). In our case, the interplay occurred during the process of selection for specialty rehabilitation within the powerful healthcare institution, which in turn is connected to the welfare system and conditions in the labour market. The ability to work as a prerequisite for rehabilitation and support is such an example.Within the context of our study, there were different ‘entrance tickets’ (related to social capital and habitus) that worked together to enhance or hinder selection and access to rehabilitation resources. The consequences of an individual being regarded as an ‘ideal patient’ (with access to specialty rehabilitation resources) or as a non-ideal patient (with access denied) were great. Outcomes were tangible for participants as they affected access to ways of coping with non-reversible pain, support, and the social health insurance system.

Equity in health is important to consider in an individual's access to healthcare and rehabilitation resources (25). According to the World Health Organization, social determinants of health are ‘mostly responsible for health inequities – the unfair and avoidable differences in health status seen within and between countries’ (54). Therefore, it is crucial to reduce inequities in global, national, and local contexts (30, 54). Swedish national public health policies emphasise equal access to health care and the decrease of inequality and inequity in the population (55). This is why it is important to scrutinise and address equal access to rehabilitation resources. Our results highlight problems and barriers in the Swedish welfare model that result in tangible consequences for some individuals.

### Methodological considerations

This study has strengths and limitations that are relevant to interpretation of the results. The strategic sample with variations in sex/gender, ages, and ethnicities is a strength (38). The fact that participants younger than 35 years were missing is a weakness. Moreover, the results may have been different if interviews were carried out separately for patients who participated in the multimodal rehabilitation programme, because they were more satisfied than the patients who were not selected to participate in the programme.

Qualitative content analysis proved to be a suitable way of exploring the wide range of perceptions, as the method keeps to a descriptive level that is close to the participants’ own words and allows the display of diversity in categories (40). Trustworthiness was obtained through thick descriptions in quotations, and interdisciplinary triangulation during the analysis (public health, physiotherapy, rehabilitation medicine, family medicine, and gender studies) (46). The fact that some of the authors had field experiences in pain and rehabilitation can be an advantage and a disadvantage. Being familiar with pain, treatment, and rehabilitation made it easier to understand the meaning and context of the interviews. On the other hand, expectations derived from pre-existing knowledge could result in overlooking descriptions. Because the authors were not acquainted with the participants, there was no possibility of their relationships influencing the interviews. The equality perspective and gender-theoretical frame functioned as a critical lens that brought additional perspectives into the analyses and data interpretation (25). It is of note that in our analysis, gender appeared to be (and was treated as) the ‘master category’. From an intersectional perspective, this is not always the case (8, 25, 31).

Despite a relatively small clinical sample with high within-group variability (e.g. gender, age, and ethnicity) and a complex research question, we believe that the study results reflect crucial aspects of perceived inequities. The results can likely be transferred to similar social contexts in terms of theoretical and analytical generalisations (46). However, there may also be diverging perceptions that were not captured in this study. Future studies need to further deepen and discuss these in relation to other rehabilitation contexts.

## Conclusion

The participants’ narrated experiences of assessment or treatment at a pain rehabilitation clinic point to perceptions of inequalities. They are formulated as the theme Accessing rehab – not a given. Potential inequalities in access to rehabilitation resources were related to gender, diagnoses, and social status. It is crucial that healthcare professionals be aware of how potential inequalities can be created during assessment or treatment. Further research is needed in this field. Knowledge of biases and inequity in the rehabilitation for long-term pain is still lacking. There is also a need for studies on how other social categories intersect and have an impact on individual's access to rehabilitation resources.

## Ethics

The study was approved by the Regional Ethics Vetting Board in Umeå, Sweden (Dnr 2010-44-31). Participants gave informed consent and were guaranteed confidentiality throughout the research process.

## Paper context

Long-term pain is common worldwide. Gender bias and inequalities in access to rehabilitation and treatment are found because of socio-economic position, age, or gender. Application of intersecting perspectives is therefore crucial. To identify potential biases in accessing rehabilitation resources, we explored patients’ perceptions from a gender and intersectional equality perspective. Access to pain rehabilitation is not perceived as a given. How to provide equal access to rehabilitation resources needs to be further scrutinised and implemented.
